# Supplementary material for: Disease Management Program in patients with type 2 diabetes mellitus, long-term results of the early and established program cohort: A population-based retrospective cohort study
Source: PLoS One. 2022 Dec 13;17(12):e0279090. doi: 10.1371/journal.pone.0279090 (PMC9746970; doi:10.1371/journal.pone.0279090)
Supplement: S1 Table — (DOCX) [file pone.0279090.s001.docx]

**S1 Table:** List of included prescriptions based on Anatomical Therapeutic Chemical (ATC) Classification System and discharge diagnoses based on International Classification of Diseases (ICD10) codes.

| **Prescriptions** | **ATC-Codes** |
| --- | --- |
| Blood glucose lowering drugs, excluding insulins | A10B |
| Insulins and analogues | A10A |
| Diuretics | C03A, C03B |
| Beta blocking agents (plain) | C07A |
| Beta blocking agents (combinations) | C07B, C07C, C07F |
| Calcium channel blockers | C08 (excluding C08CA06) |
| ACE inhibitors (plain) | C09A |
| ACE inhibitors (combinations) | C09B |
| Angiotensin II antagonists (plain) | C09C |
| Angiotensin II antagonists (combinations) | C09D |
| Renin inhibitors | C09X |
| HMG-CoA reductase inhibitors | C10AA |
| Lipid modifying agents (combinations) | C10B |
| Other lipid modifying agents | C10AB, C10AC, C10AD, C10AX |
| Psycholeptics | N05 |
| Psychoanaleptics | N06A, N06C |
| Analgesics | N02 |
| Anti-inflammatory and antirheumatic products | M01A, M01B |
| **Discharge diagnoses** | **ICD10-codes** |
| Diabetes mellitus | E10-E14 |
| Obesity | E66 |
| Other polyneuropathies | G62 |
| Polyneuropathy in diseases classified elsewhere | G63 |
| Disorders of choroid and retina | H30-H36 |
| Disorders of vitreous body | H43 |
| Hypertensive diseases | I10-I15 |
| Ischemic heart diseases | I20-I25 |
| Other forms of heart disease | I42-I52 |
| Cerebrovascular diseases (without I60) | I61-I69 |
| Diseases of arteries, arterioles and capillaries | I70-I79 |
| Glomerular disorders in diseases classified elsewhere | N08 |
| Renal failure | N17-N19 |
| Gangrene, not elsewhere classified | R02 |
| Traumatic amputations (feet/legs) | T05.3-T05.5 |
| Traumatic amputation of lower limb, level unspecified | T13.6 |
