# Supplementary material for: Disease Management Program in patients with type 2 diabetes mellitus, long-term results of the early and established program cohort: A population-based retrospective cohort study
Source: PLoS One. 2022 Dec 13;17(12):e0279090. doi: 10.1371/journal.pone.0279090 (PMC9746970; doi:10.1371/journal.pone.0279090)
Supplement: S2 Table — (DOCX) [file pone.0279090.s002.docx]

**S2 Table: Established program cohort:** Descriptive statistics for the DMP-group and the control-group before and after matching.

| **Matching parameters** | **DMP-group**  **N=3087** | | **Control-group** | | | |
| --- | --- | --- | --- | --- | --- | --- |
|  |  |  | **before matching** | | **after matching** | |
|  |  |  | **N=117062** | | **N=9261** | |
|  | **N**  **mean (SD)** | **%**  **median**  **(min-max)** | **N**  **mean (SD)** | **%**  **median**  **(min-max))** | **N**  **mean (SD)** | **%**  **median**  **(min-max)** |
| sex |  |  |  |  |  |  |
| female | 1482 | 48.0 | 55974 | 47.8 | 4402 | 47.5 |
| male | 1605 | 52.0 | 61088 | 52.2 | 4859 | 52.5 |
| age | 64 (12) | 64 (18-95) | 68 (11) | 69 (18-99) | 64 (12) | 64 (18-97) |
| prescription fee | 1397 | 45.3 | 58235 | 49.7 | 4188 | 45.2 |
| hospital days |  |  |  |  |  |  |
| none | 2042 | 66.1 | 76196 | 65.1 | 6122 | 66.1 |
| 1-7 days | 541 | 17.5 | 19004 | 16.2 | 1584 | 17.1 |
| 8-14 days | 227 | 7.4 | 9130 | 7.8 | 718 | 7.8 |
| 15-30 days | 170 | 5.5 | 7689 | 6.6 | 567 | 6.1 |
| >30 days | 107 | 3.5 | 5043 | 4.3 | 270 | 2.9 |
| hospital days >0 | 12.7 (16.1) | 7 (1-171) | 14.6 (19) | 8 (1-391) | 12.4 (15.6) | 7 (1-235) |
| total costs (€) | 3122 (4712) | 1636  (15-90894) | 3621 (5485) | 1881  (7-193560) | 3111 (4427) | 1709  (11-106900) |
| therapy form |  |  |  |  |  |  |
| none | 444 | 14.4 | 10814 | 9.2 | 1343 | 14.5 |
| OAD only | 2093 | 67.8 | 82324 | 70.3 | 6304 | 68.1 |
| Insulin only | 233 | 7.5 | 10291 | 8.8 | 687 | 7.4 |
| combination | 317 | 10.3 | 13633 | 11.6 | 927 | 10 |
| prescriptions |  |  |  |  |  |  |
| C03A, C03B | 107 | 3.5 | 5182 | 4.4 | 307 | 3.3 |
| C07A | 1054 | 34.1 | 44323 | 37.9 | 3163 | 34.2 |
| C07B, C07C, C07F | 217 | 7 | 8450 | 7.2 | 655 | 7.1 |
| C08 (except C08CA06) | 691 | 22.4 | 30245 | 25.8 | 2119 | 22.9 |
| C09A | 809 | 26.2 | 33138 | 28.3 | 2469 | 26.7 |
| C09B | 794 | 25.7 | 30607 | 26.1 | 2406 | 26 |
| C09C | 359 | 11.6 | 14394 | 12.3 | 1074 | 11.6 |
| C09D | 551 | 17.8 | 21881 | 18.7 | 1687 | 18.2 |
| C10AA, C10B | 1561 | 50.6 | 62827 | 53.7 | 4667 | 50.4 |
| C10-others | 137 | 4.4 | 5630 | 4.8 | 437 | 4.7 |
| N05 | 394 | 12.8 | 18262 | 15.6 | 1191 | 12.9 |
| N06A, N06C | 680 | 22 | 25964 | 22.2 | 2019 | 21.8 |
| N02 | 620 | 20.1 | 24211 | 20.7 | 1819 | 19.6 |
| M01A, M01B | 1422 | 46.1 | 52058 | 44.5 | 4286 | 46.3 |
| discharge diagnosis total | 438 | 14.2 | 17151 | 14.7 | 1358 | 14.7 |
| discharge diagnosis |  |  |  |  |  |  |
| E10-E14 | 162 | 5.2 | 5524 | 4.7 | 491 | 5.3 |
| E66 | 10 | 0.3 | 269 | 0.2 | 31 | 0.3 |
| G63 | 1 | 0 | 117 | 0.1 | 10 | 0.1 |
| G62 | 4 | 0.1 | 71 | 0.1 | 4 | 0 |
| H30-H36 | 42 | 1.4 | 1632 | 1.4 | 130 | 1.4 |
| H43 | 3 | 0.1 | 104 | 0.1 | 5 | 0.1 |
| I10-I15 | 49 | 1.6 | 1926 | 1.6 | 166 | 1.8 |
| I20-I25 | 104 | 3.4 | 3700 | 3.2 | 291 | 3.1 |
| I42-I52 | 76 | 2.5 | 3328 | 2.8 | 212 | 2.3 |
| I61-I69 | 33 | 1.1 | 1562 | 1.3 | 121 | 1.3 |
| I70-I79 | 49 | 1.6 | 1738 | 1.5 | 119 | 1.3 |
| N08 | 0 | 0 | 3 | 0 | 0 | 0 |
| N17-N19 | 12 | 0.4 | 834 | 0.7 | 49 | 0.5 |
| R02 | 3 | 0.1 | 138 | 0.1 | 6 | 0.1 |
| T05.3-T05.5 | 0 | 0 | 1 | 0 | 0 | 0 |
| T13.6 | 0 | 0 | 1 | 0 | 0 | 0 |
| hospital admissions |  |  |  |  |  |  |
| 0x | 2042 | 66.1 | 76196 | 65.1 | 6122 | 66.1 |
| 1x | 587 | 19 | 21476 | 18.3 | 1769 | 19.1 |
| 2x | 245 | 7.9 | 9668 | 8.3 | 721 | 7.8 |
| >2x | 213 | 6.9 | 9722 | 8.3 | 649 | 7 |
| hospital admissions >0 | 1.9 (1.5) | 1 (1-16) | 2.1 (2.1) | 1 (1-83) | 2 (2.5) | 1 (1-83) |
