# Supplementary material for: Disease Management Program in patients with type 2 diabetes mellitus, long-term results of the early and established program cohort: A population-based retrospective cohort study
Source: PLoS One. 2022 Dec 13;17(12):e0279090. doi: 10.1371/journal.pone.0279090 (PMC9746970; doi:10.1371/journal.pone.0279090)
Supplement: Attachment — Submitted filename: Response to Reviewers.docx [file pone.0279090.s003.docx]

**RESPONSE LETTER**

[PONE-D-22-21147] - [EMID:650aa889ffb549a1]

Disease Management Program in patients with type 2 diabetes mellitus, long-term results of the early and established program cohort: a population-based retrospective cohort study

PLOS ONE

Dear Editors;

Dear Reviewers!

Thank you for giving us the opportunity to submit a revised version of our manuscript “Disease Management Program in patients with type 2 diabetes mellitus, long-term results of the early and established program cohort: a population-based retrospective cohort study”. We also thank the reviewers for their useful comments. We provide one clean revised version and a version with tracked changes so you can see where the changes have been made. For our point-by-point response, in blue, see below.

We hope that we have responded to your satisfaction, and that you will now find our paper suitable for publication in PLOS ONE.

On behalf of the co-authors,

Sincerely,

Andrea Berghold

# Reviewer #1:

Thank you submitting this interesting paper exploring the effect of the DMP program on mortality, diabetes-related complications, costs and process quality of care parameters in the Austrian T2DM population.

The paper is overall well written and worth of attention, as highlighting the importance of dedicated care and dedicated programs to tackle diseases (T2DM in this specific case) that nowadays still present with incredibly high morbidly and mortality rates notwithstanding the extensive research in both terms of prevention and treatment.

In my opinion, however, the lack of data concerning disease duration and values of HbA1 values represents a strong limitation to the interpretation of results. In fact, even if the improvement in general care may explain the reduction in mortality and diabetes-specific complications HR in the control group, this does not fully explain why figures do not show the same trend in the DMP group as well. Could this be explained by patients enrolled in the DMP program being in more advanced stages of disease or having more difficulties in controlling DM parameters, as a consequence being more motivated in taking part to the program? In this scenario, the beneficial effect of the program may be not extended to the whole population but, on the contrary, to a specific part of it with specific inclusion criteria and the adherence to program itself be biased by baseline clinical conditions.
I would maybe consider and expand this point more.

We thank the reviewer for the positive feedback. We agree that the lack of clinical data is a strong limitation of our study and we already stated our concerns about the generalizability of our results and possible unmeasured confounding in the discussion section. In order to point this out even more clearly, we have added the following parts to the discussion:

“*However, mortality in the DMP group remained similar across the cohorts included in the matchings. Similar baseline characteristics were observed as well. Due to the lack of data on disease duration and HbA1c measurements, it cannot be ruled out that the cohorts might differ in disease severity, which could explain the lack of improvement across the DMP cohorts.*”

“A further limitation represents the fact that important clinical parameters like HbA1c measurements and duration of diabetes were not available. This might affect the comparability of our groups *due to different baseline clinical conditions* (unmeasured confounding), and allows only general statements about effectiveness of the program. *It might be possible that patients with more advanced disease stage and/or having more difficulties in controlling diabetes relevant clinical parameters are included in the DMP group of the established program cohort. However, due to the lack of clinical data, this cannot be verified.*”

# Reviewer #2:

In this observational case-control study, the authors investigate the effectiveness of the Disease Management Program “Therapie aktiv – Diabetes im Griff”, a systematic treatment program for patients with diabetes mellitus type 2. They investigate the long-term effects of the program for participants enrolled in the early phase (2008-2009) and evaluated a cohort enrolled later, in the established phase of the program, adding patient-relevant outcomes (overall mortality, cardiovascular disease) and quality of care.

The main findings are that patients included in the early phase of the program have lower mortality rates and lower health-related costs, mainly due to the lower rate of hospitalizations. Patients newly included had lower mortality and a better quality of care compared with controls, but the costs were similar. In particular, the authors observe that mortality remained similar in patients enrolled in the program (both early phase and established phase), but in the control groups, which are different for the two cohorts, mortality decreased significantly.

The manuscript reports interesting results, however some concerns should be addressed:

- It would be useful for the reader to have a more detailed description of the program and what it encompasses. How are patients enrolled and how do physicians choose to participate?

We described the program in more details in the Introduction of the manuscript and added a link to the homepage of the program (<http://www.therapie-aktiv.at>) as well. The following paragraph was added:

*“A basic training for physicians based on evidence-based clinical guidelines is necessary before they can work as DMP-physicians. Key features of the program include: necessary medical examinations on a regular basis, patient empowerment (by definition of individual target agreements by patient and the physician), lifestyle advice (e.g. change in diet habits and physical activity), and regular medical documentation by DMP-physicians (medical parameters, treatment, target agreements and quality of life).”*

We updated the number of DMP participants and physicians as well.

- The results do not seem to be thoroughly discussed. There is no reference to similar programs in other countries. While they might be quite different because of diverse healthcare systems, it might be meaningful to discuss the goal of such programs.

We have added a new paragraph in our discussion section where we discussed already referenced literature more thoroughly and furthermore included the following two references:

[35]: Fuchs S, Henschke C, Blümel M, Busse R. Disease management programs for type 2 diabetes in Germany: a systematic literature review evaluating effectiveness. Dtsch Arztebl Int. 2014; 111(26): 453–63. doi: 10.3238/arztebl.2014.0453 PMID: 25019922

[36]: Höglinger M, Wirth B, Carlander M, Caviglia C, Frei C, Rhomberg B, Rohrbasser A, Trottmann M, Eichler K. Impact of a diabetes disease management program on guideline-adherent care, hospitalization risk and health care costs: a propensity score matching study using real-world data. Eur J Health Econ. 2022 Jun 18. doi: 10.1007/s10198-022-01486-2. Epub ahead of print. PMID: 35716315.

New paragraph in Discussion:

*“The main goal of implemented DMPs in Europe is to improve the quality of chronic healthcare and thereby improve patient outcomes including quality of life and lower diabetes-related complications and extend their lifetime in good health. A comprehensive overview of the key features of DMPs for diabetes and their evaluations in Europe is given in Kostial et al. [6]. Due to large heterogeneity in evaluation methodology, direct comparisons of the results are difficult. However, similar to the DMP in Austria is the DMP in Germany [15]. Beneficial impact on mortality but inconsistent effects on morbidity, quality of life and economic parameters were observed by Fuchs et al. [35] in earlier program evaluations. More recently, supporting our results for process quality of care, Mehring et al. [10] observed an improvement in quality of care, patient education and therefore improved adherence to guidelines over time in diabetic DMP participants in Germany. A better quality of care was also observed by Höglinger et al. [36] who investigated the impact of DMPs in Switzerland on similar quality of care parameters as in our study. In their evaluation, patients with diabetes mellitus type 1 and type 2 and treated with antidiabetic medication were included and better guideline-adherent care (including measurement of HbA1c, lipid profile, nephropathy status and examinations by the ophthalmologist) was observed in a follow-up period of two years.”*

- How were diabetes complications chosen? Macrovascular disease seems to be quite well represented but kidney disease and retinopathy are not included. Moreover, one could evaluate including heart failure hospitalizations, as they presumably have a significant impact on health costs and might be related to poor diabetes control.

To be able to achieve highly reliable results for our Austrian real world data we have chosen the major macrovascular diabetes complications, myocardial infarction and stroke. As in our first evaluation (Riedl et al [15]) we focused on these complications because of data quality issues – in the LEICON database only the admission diagnosis and two discharge diagnoses from hospital stays are documented and no standardized coding of diagnoses with ICD-10 in the outpatient sector is performed – and frequency of these complications. We changed the wording in the manuscript to be more specific regarding the diabetic complications chosen.

In the established program cohort we also identified another severe macrovascular-related complication, namely amputations which were extracted based on individual medical procedure groups. These data were not available in the first evaluation.

- While it is understandable that one uses PS matching to investigate outcomes, in a study having a descriptive part one might be interested in the comparison of patients included in the program with those not included in general. Which are the factors that make it more likely to be included vs not included?

For the early program cohort evaluation, detailed information on baseline characteristics for patients not included in the program in general (i.e. before matching) are already published in Riedl et al [15], Table S3. We now included a brief description of differences in the groups before matching in the current manuscript.

*“Detailed characteristics of the DMP and control-group before and after matching are published in Riedl [15]. Before matching, patients in the control group tended to be older with a higher number of hospital days and total costs compared to DMP participants.”*

For the established program cohort, characteristics before and after matching are presented in supplemental materials (Table S2). Baseline group differences were observed for the same parameters, although the differences were slightly less pronounced. In the manuscript, we added:

*“Before matching, group differences in age (controls were older), number of hospital days and total costs (higher in controls) were observed.“*

Minor issues:

- Mismatch in references: for example, it is stated that Riedl et al is ref 15 whereas it is ref 16

Thank you for bringing this to our attention. The numbering of the references was wrong and was now changed accordingly.

- References 1 and 2 should be updated with new data form more recent reports

We have now included recent literature.
